# Gingival Crevicular Fluid Peptidome Profiling in Healthy and in Periodontal Diseases

**DOI:** 10.3390/ijms21155270

**Published:** 2020-07-24

**Authors:** Mariaimmacolata Preianò, Rocco Savino, Chiara Villella, Corrado Pelaia, Rosa Terracciano

**Affiliations:** 1 Department of Health Sciences, University “Magna Græcia”, 88100 Catanzaro, Italy; preiano@unicz.it (M.P.); villella@unicz.it (C.V.); pelaia.corrado@gmail.com (C.P.); 2 Department of Medical and Surgical Sciences, University “Magna Græcia”, 88100 Catanzaro, Italy; savino@unicz.it; 3 Department of Experimental and Clinical Medicine, University “Magna Græcia”, 88100 Catanzaro, Italy

**Keywords:** gingival crevicular fluid, MALDI-TOF, peptides, antimicrobial peptides, peptidomics, periodontal diseases, mass spectrometry

## Abstract

Given its intrinsic nature, gingival crevicular fluid (GCF) is an attractive source for the discovery of novel biomarkers of periodontal diseases. GCF contains antimicrobial peptides and small proteins which could play a role in specific immune-inflammatory responses to guarantee healthy gingival status and to prevent periodontal diseases. Presently, several proteomics studies have been performed leading to increased coverage of the GCF proteome, however fewer efforts have been done to explore its natural peptides. To fill such gap, this review provides an overview of the mass spectrometric platforms and experimental designs aimed at GCF peptidome profiling, including our own data and experiences gathered from over several years of matrix-assisted laser desorption ionization/time of flight mass spectrometry (MALDI-TOF MS) based approach in this field. These tools might be useful for capturing snapshots containing diagnostic clinical information on an individual and population scale, which may be used as a specific code not only for the diagnosis of the nature or the stage of the inflammatory process in periodontal disease, but more importantly, for its prognosis, which is still an unmet medical need. As a matter of fact, current peptidomics investigations suffer from a lack of standardized procedures, posing a serious problem for data interpretation. Descriptions of the efforts to address such concerns will be highlighted.

## 1. Introduction

### 1.1. Gingivitis and Periodontitis

Periodontal diseases are complex, multifactorial, highly prevalent chronic inflammatory disorders affecting the tooth supporting tissues. Among them, periodontitis is a complex immune–inflammatory disease representing a significant health issue due to its high prevalence worldwide with approximately 10–15% of individuals being affected by severe forms [1]. It is characterized by microbially-associated, host-mediated inflammation that, in the absence of advanced treatment, could lead to a loss of periodontal attachment and also to alveolar bone resorption, resulting in deep periodontal lesions that may cause significant tooth loss in the advanced stage of pathology [2]. The accumulation of activated inflammatory cells like polynuclear leukocytes, macrophages, and lymphocytes in response to dental plaque amplifies the inflammatory process by the release of pro-inflammatory mediators [3]. Instead, gingivitis is recognized as a reversible condition, associated with microbial plaque accumulation and gingival inflammation that often resolves after restoration of oral hygiene procedures but, if untreated, may ultimately progress to periodontitis, thus representing the transitional phase between health and periodontal disease [1]. Therefore, a crucial interest has been growing in delineating the mechanism underlying this inflammatory stage, before it evolves to periodontitis. Periodontitis diagnosis is based on clinical examination, medical and dental history, tooth mobility, and radiographic assessment. Clinical evaluation is based on the assessment parameters, such oral hygiene, gingival status, probing depth, bleeding on probing, clinical attachment loss, alveolar bone status, but also on procedures such as periodontal microbiology testing, systemic health profiling by blood analysis, and finally, histological studies [4]. The main limit of most of these clinical methods is a certain degree of subjectivity, since they are examiner dependent. In addition, the biological events which trigger the progression from health to disease conditions are still unclear, therefore there is an urgent need to discover and validate reliable biomarkers able to diagnose early stage of pathology, possibly before clinical manifestations of periodontitis, in order to allow a more effective prompt therapeutic intervention.

Diagnostic tests are generally not necessary to discriminate between an inflamed site and a healthy site because clinical indices of inflammation, determined as described above, correspond well enough with periodontium histological inflammation. However, from a diagnostic point of view, the ability to discriminate between an inflamed-progressive (active) site and a less critical inflamed but stable site is of paramount importance [5,6]. Now, it is well accepted that both the outcomes of the immune/inflammatory response present in saliva/gingival crevicular fluid (GCF) and the impact of behavioral and environmental factors deeply affect the clinical picture of periodontopathy [7]. Following the “omics” burst, many researchers have tried to unravel molecular markers of periodontitis, identifying several genes, transcripts, proteins, and metabolites related to the pathology. Therefore, in the attempt to screen molecular profiles of periodontal disease, GCF has gained much attention, as will be discussed in the following sections.

### 1.2. Nature and Function of GCF

GCF is released in the dental crevice or gingival sulcus, defined as the space between the tooth and the overlying unattached gingiva. It mirrors serum composition because it not only derives from the gingival plexus of blood capillaries in the gingival corium, but also contains molecules derived from both host tissues and subgingival plaque, as well as a number of different cells, including epithelial cells, leukocytes and bacteria from the adjacent plaque mass [8]. In contrast, specialized glands, in particular the major salivary glands (submandibular, sublingual and parotid glands), and also roughly 600 minor salivary glands that are dispersed throughout the oral mucosa, have evolved to secrete saliva in the mouth. Obviously, GCF flows from the dental crevice into the buccal cavity, mixing with saliva and becoming part of it. Therefore, saliva is an aqueous solution (water content of 99%) containing electrolytes, small organic molecules, and nucleic acids, but most of all, proteins. In fact, over 3000 proteins have been found in saliva, the most represented being mucins, amylases, proline-rich proteins, cystatins, statherin, and histatins [4].

GCF exists as a serum transudate, changing into an inflammatory exudate as the inflammatory events progress [4]. In fact, this fluid is described as a mixture of molecules derived from the blood, host tissues, and subgingival plaque, and it contains electrolytes, small organic molecules, and various kind of proteins, among which antibodies, cytokines, bacterial antigens, and finally, enzymes of both host and bacterial origin [9]. Cytological examination of the GCF also demonstrates that it contains a variety of different cell types, including bacteria from the neighboring plaque mass, desquamated epithelial cells (which are passively washed out of the dental crevice into the oral cavity by the outward GCF flow), and transmigrating leukocytes (among which, lymphocytes monocytes/macrophages, and polymorphonuclear cells). Erythrocyte detection in the GCF should be considered an auxiliary finding, indicating injury to the microvessels that is typical of progressive inflammatory process [10]. With the onset of inflammation, the GCF flow rate increases and its composition begins to look like that of an inflammatory exudate. The augmented GCF flow contributes to host immune defense by flushing both bacteria and their metabolites away from the dental crevice. The main route for the diffusion of GCF is through the basement membrane, and subsequently, through the junctional epithelium into the dental crevice [11]. A particularly attractive feature of GCF is that this fluid is “periodontal specific” as it stems from a local site deeply involved in the manifestation of periodontal disease. For this reason it is considered to represent a faithful mirror of the periodontal health state of an individual [4]. Due to its composition, GCF might be a source of new biomarkers of periodontitis/gingivitis. The oral cavity is indeed a reserve of the microbiome and, when adverse changes occur within the oral cavity, it results in pathological changes, such as gingivitis, periodontitis, and dental caries [8]. As a biomonitoring fluid, GCF may play a key role both in the diagnosis and in the prognosis of oral diseases, in particular for periodontal diseases. It can be included among the most non-traumatic proximal fluid to obtain information about periodontal tissue status, including the conditions of the connective tissue and the level of hard destruction [12]. A non-invasive GCF collection (paper strip, paper points) technique helps obtaining samples of all age groups of human subjects, allowing also multiple sites sampling within the oral cavity [13]. So far, diverse inflammatory mediators have been isolated from GCF, including cytokines, phosphatase, proteinase and local tissue degradation products [14]. Several proteomics studies in the last decade shed light on GCF as potential source of biomarkers of both gingivitis and periodontitis and provided evidence for its diagnostic usefulness. The differential expression level of several proteins, such as defensins, annexins, plastin-2, lipocalin-2, and S100-P, was reported in the GCF between health and disease [15].

### 1.3. Low Molecular Weight Profiling of GCF

Despite the fact that naturally occurring peptides present in bodily fluids are known to play both local and/or systemic functions, only a few proteomics/peptidomics investigations targeted the GCF peptidome [6,16,17,18,19,20,21,22,23,24,25,26,27,28,29]. Even with such limited studies, the knowledge of the biological mechanisms that can be gained from peptidomics applied both to the identification of markers of disease and to the development of therapies which has encouraged the use of in the context of progress made in this field [6,16,17,18,19,20,21,22,23,24,25,26,27,28,29]. In the last decade, the impact of proteomics to the understanding of human GCF composition has progressively increased, underlining the potential diagnostic role of this fluid as invaluable source of periodontal disease-specific markers [6,14,16,17,18,19,20,21,22,23,24,25,26,27,28,29,30,31,32,33,34,35,36,37,38,39,40,41,42,43,44,45,46,47].

**Scheme 1:**
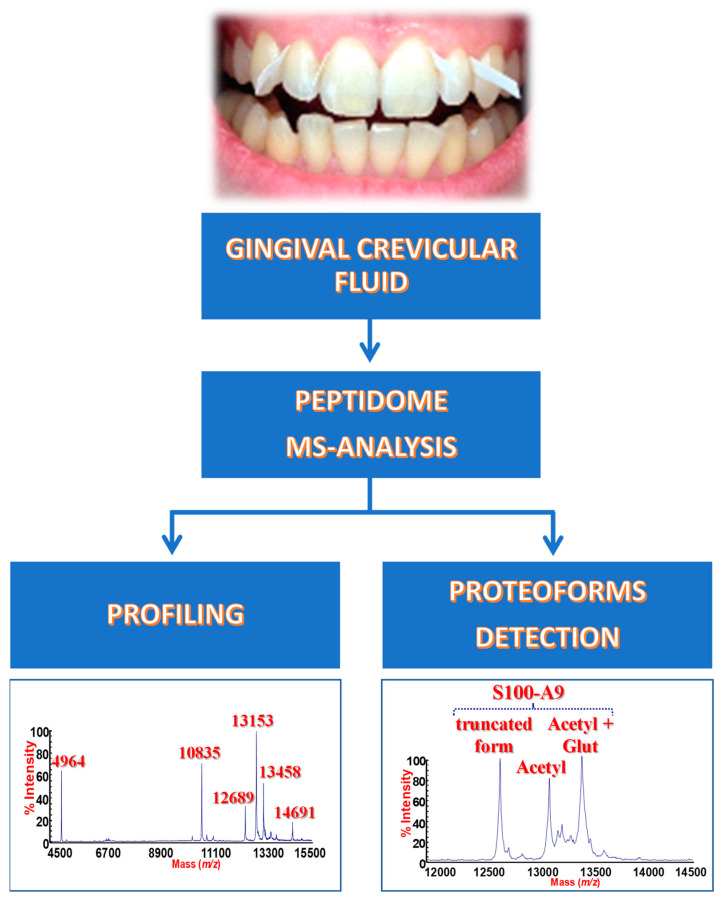
Overview of the main informational data extrapolated by peptidomics experiments on gingival crevicular fluid (GCF). Peptidomics workflow, without a digestion step, allows to preserve the endogenous information of peptides from GCF, including post-translational modifications and proteolytic products.

With the progress of bottom-up MS-based approaches, enormous amounts of data have been generated, especially concerning the protein content of GCF, and extraordinary attention has been paid to comparative analysis in order to identify differentially expressed proteins between healthy and periodontal disease [48,49]. While from one point of view, it appears remarkable to categorize these studies as based on discovery or on quantitative proteomics approaches, as recently outlined by both Bostanci and colleagues [15,48] and Tsuchida and collaborators [49], it could also be of interest to appraise the efforts done by the proteomics community to assess the invaluable role played by naturally occurring peptides in GCF. Therefore, in the present review we highlight peptidomics studies which offer significant opportunities to detect and to identify peptides and small proteins in GCF of interest for periodontal diseases (Scheme 1, Table 1 and Table 2). A special focus will be given to the study design of several investigations, which are described from the collection of the samples to their processing and to the MS platform and strategy adopted, all with the aim to better address current limitations which hurdle GCF peptidomics research. Concerning the methodological strategy, all the studies reviewed here were carried out with MS profiling approaches in which protein content of GCF did not undergo protease digestion before MS analysis. The absence of a digestion step preceding the MS analysis overcome the intrinsic limitation of not discriminating between the naturally occurring peptides found in GCF and those artificially generated by proteolysis in the preceding step required for a bottom up protocol. The relevance of the top down profiling approaches reviewed here resides therefore in the fact that such investigations might reveal in a more direct and clear manner specific proteins/peptides functions and mechanisms of actions, as top down approaches preserve valuable information about all of proteoforms including proteolytic cleavage from larger proteins by the action of proteases of human or bacterial origin.

Generally, the peptide profiling investigations that we summarize in this review make use of surface enhanced laser desorption ionization (SELDI) [17,18] and matrix-assisted laser desorption ionization (MALDI)-MS [6,19,20,21,22,23,24,25,26] approaches which result more suitable and easily applicable for the analysis of natural peptides of GCF (Table 1). However, few investigations based on electrospray ionization (ESI)-MS have also been carried out to explore peptidic components and small proteins found in GCF in a top-down fashion [27,28,29] (Table 1).

.

## 2. An Overview of MS Profiling Tools and Investigations to Detect GCF Peptides

Some, but not many investigations based on MS have been performed to explore the GCF peptidome both under physiological [20,22,23,27,28] and pathological conditions [6,16,17,18,19,21,24,25,26,29]. Targeted identifications of specific peptidic component in GCF have been reported by Lundy et al. [16]. In this study, Calgranulin A (S100-A8) was purified by high-performance liquid chromatography (HPLC) and then identified using N-terminal amino-acid sequencing. S100-A8 is expressed in cells of myeloid origin and secreted in the case of cell damage or activation of phagocytes and monocytes. It exerts many roles in periodontal inflammation including chemotaxis, phagocytosis, or inhibition of microbial growth [16]. The amount of Calgranulin A was determined in GCF from healthy, gingivitis and periodontitis sites both in periodontitis patients (*n* = 15) and also from controls (*n* = 5) with healthy gingival status. The results showed significant differences in the amounts of Calgranulin A in relation to disease status, with higher expression levels in periodontitis and gingivitis sites than in the healthy sites in both periodontitis subjects and controls (Table 2) [16].

### 2.1. SELDI MS GCF Peptidome Profiling

In SELDI MS, samples to be analyzed are applied directly to a biochip coated with specific chemical “bait” matrices, which may be comprised of supports routinely used in chromatographic techniques (anionic, cationic, hydrophobic surfaces, etc.) or biochemical bait molecules, for instance purified ligands, proteins (like antibodies, receptors) or also DNA oligonucleotides [50]. The protein population bound to the biochip can then be washed with different kinds of washing buffers, in such a way that only the proteins sharing common chemical features remain on the surface and, subsequently, the mass map of all proteins retained is obtained at the same time. This analysis gives rise to a protein “fingerprint” formed by the exact molecular weight of each and every single protein first bound and then ionized off the specific bait surface used. SELDI-time of flight (TOF) MS, is one of the best analytical techniques for profiling both peptides and also low MW proteins (<20 kDa). Indeed, it has been extensively used in proteomics studies and, due to its high throughput, it became a promising tool for the identification of diagnostic patterns of disease [51,52,53]. However, this technology suffers a number of drawbacks preventing its widespread application as a routine platform in clinical diagnostics [54]. One of the main disadvantages of SELDI-TOF MS in peptidomics analysis is that its resolution is generally not very high (i.e., several different peptides can be present within each single ion peak, thus preventing direct assignment of the ion peaks of interest) [55]. Another point of weakness of the platform is the poor reproducibility, both within and also between laboratories. In this respect, a number of efforts have already been made to set up standardized protocols among the various research groups [56]. Another limitation of SELDI-TOF instruments is that they are not able to directly identify putative biomarkers as they lack MS/MS capabilities [57]. Finally, concerns can also be risen related to both the sensitivity of the technique and also the specificity of the biomarkers discovered by SELDI analysis [54].

The first peptidomics study on GCF was pioneered by Diamond and colleagues [18]. With the aim to understand the physiologic role of beta-defensins in the oral environment, Diamond and colleagues examined the media of cultured human oral keratinocytes and GCF by ProteinChip Array SELDI-TOF MS technology. The authors found that both the 47-amino acid form of human β-Defensin 1 (hBD-1, *m*/*z* = 5069) and the 41-amino acid form of human β-Defensin 2 (hBD-2, *m*/*z* = 4330) were best detected in GCF by SELDI-TOF measurements when a chip (among the others) coated with a hydrophilic phase was used. Peptides with the predicted masses of the alpha-defensins HNP-1 (*m*/*z* = 3442), HNP-2 (*m*/*z* = 3371) and HNP-3 (*m*/*z* = 3486) were also detected. However, only the identity of hBD-1 and hBD-2 was indirectly confirmed by immunoaffinity capture with anti-hBD-1 and anti-hBD-2 polyclonal antibodies on the ProteinChip surface, although hBD-1, as the authors hypothesized, was found in several smaller forms suggesting extracellular proteolysis. Immunoaffinity capture experiments unmasked inter-individual variation between the two subjects (*n* = 2) in the relative amount of hBD-2 and the hBD-1 peptides. Specifically, more hBD-2 and alpha-defensins, with little hBD-1, was seen in the case of the subject with greater inflammation. β-Defensins are secreted by gingival epithelial cells and exert combined antimicrobial, chemotactic, and anti-inflammatory properties [18]

With the same technology, some years later, Dommisch and colleagues optimized the experimental conditions to obtain the simultaneous detection of human neutrophil antimicrobial peptides (AMPs) in GCF as well as human cathelicidin LL-37 in SELDI-TOF profiles [17]. LL-37 is secreted by neutrophils and plays a key role in innate immune responses [17]. In this study, they compared GCF specimen from healthy periodontal sites to those from sites with early gingival inflammation. As each single donor also provided GCF samples from healthy periodontal sites as an internal control, GCF samples from sites diagnosed as gingivitis were analyzed individually in a donor-specific manner, thus allowing relative quantification. The authors observed that donors (four out of eight donors) that showed significantly higher intensity peaks corresponding to the mass of LL-37 (*p* = 0.01) also displayed greater (although not statistically significant) intensity peaks corresponding to the molecular masses of HNP-1 and HNP-2 in samples obtained from inflamed as compared to healthy periodontal sites (see Table 2). However, also in this SELDI study, the main limitations are due to the loss of unambiguous sequence-based identifications of the putative *m*/*z* peaks detected in the mass spectra and to the inherent semi quantitative assay.

### 2.2. MALDI MS GCF Peptidome Profiling

In the MALDI-TOF MS, a large excess of matrix (which is a small organic aromatic UV absorbing molecule) is used in comparison to the amount of the analytes (in this case peptides that need to be analyzed). Peptides are co-crystallized with the matrix into a solid-phase onto a stainless-steel target plate. When a laser beam hits the surface of the solid mixture, the matrix absorbs the laser energy and transfers it to the protonated peptides. Simultaneously, the rapid heating determines desorption of both the matrix and the newly formed [M + H]^+^ protonated peptides directly into the gas phase. MALDI ion source is commonly coupled to TOF mass analyzer [58]. In the recent mass analyzers, the ions generated in the source are then accelerated to a fixed amount of kinetic energy and travel along a flight tube. Having all the same kinetic energy, the small ions have a higher velocity and are therefore recorded by a detector before the larger ones. As a consequence, each *m*/*z* value displayed in a TOF spectrum is proportional to the time required to reach the detector for each given analyte.

Specifically, MALDI-TOF MS is a very versatile and fast analytical platform for analyzing both small and large polypeptides contained in various kind of biological samples. Some intrinsic properties of this analytical technique include low sample consumption, simple sample preparation, remarkable tolerance toward salts and buffers, high degree of automation and high throughput analysis and, finally, high sensitivity and mass accuracy. All these features make MALDI-TOF MS highly suitable for diagnostic purposes [59,60,61,62,63]. The versatility of the MALDI-TOF MS platform was exploited in different approaches to analyze GCF peptidome [6,19,20,21,22,23,24,25,26].

Lundy and colleagues used MALDI-TOF MS to qualitatively analyze neutrophil alpha-defensins in unfractionated GCF samples [19]. Starting from the rationale that the triad of alpha-defensins peptides (HNP-1, HNP-2, and HNP-3), due to their similar primary sequence, might show an equal ionization efficiency during the MALDI-TOF MS measurements, these investigators assumed that the ion intensities of the peaks representing the alpha-defensins paralleled the relative amount of these peptides. Therefore, they assessed the relative intensity of the peaks corresponding to HNP-1, HNP-2 and HNP-3 detected in the MALDI-TOF mass spectra of the unfractionated GCF collected from 12 periodontally healthy and 11 periodontally diseased subjects. From a comparison of the relative abundance of defensins, they found that the peak corresponding to the mass of HNP-1 was always the highest of the triplet while the peak matching with HNP-3 was always the lowest. These findings are consistent with the speculation that HNP-2 could be produced selectively by proteolysis of HNP-3, but not of HNP-1 [64]. Additionally, on the basis of ion intensity scores, the authors observed that the defensins were more abundant in a higher proportion of the healthy sites in comparison to the diseased (although the differences were not statistically significant). Therefore, they hypothesized that high expression of HNPs in GCF of periodontally healthy gingivae serves as barrier against bacterial colonization. These findings on defensins expression based on MALDI-MS data from Lundy [19] were however in contrast with those observed later by Dommisch [17] in the SELDI-based investigations (Table 2).

Wen et al. performed a profiling proteomic approach using GCF from 20 pubertal and 20 post-pubertal subjects in order to identify novel candidate biomarkers in GCF useful for the diagnosis of pubertal growth peak [20]. The GCF samples were collected by paper points and stored at −80 °C until MALDI-TOF analysis. Sample processing was performed using TFA, without the use of a protease inhibitor cocktail (PIC) (see Table 1). The unfractionated samples were analyzed directly by MALDI-TOF MS. The statistical analysis was performed after normalization for peak intensity and the results showed that six peptides were significantly different between the two groups. In particular, the peaks at *m*/*z* 1660.2, 1783.0 and 6108.9 were upregulated in the pubertal group compared to the post-pubertal group, while the peaks at *m*/*z* 5064.9, 2912.5 and 4178.6 were downregulated in the pubertal group compared to the post-pubertal group. Although primary sequence of these potential candidate biomarkers has not been identified, still the study provided a statistical model based on MALDI-TOF MS data able to detect the pubertal growth peak, demonstrating the potential use of this approach as a periodontal diagnostic tool. It should be taken into account that normalization for protein concentration was not performed, nor was the time of storage at −80 °C indicated.

Ngo and colleagues were the first to apply the sequencing capability of MALDI-TOF/TOF MS to identify the endogenous peptides of GCF collected from inflamed sites [6]. By means of this technology, 33 endogenous peptides were identified with a molecular weight ranging from 1067 Da to 3700 Da. In particular, of these 33 peptides, 10 peptides were identified in unfractionated GCF from single sites from direct TOF/TOF sequencing experiments while the other 23 peptides were identified in GCF samples pooled from multiple inflamed sites and fractionated by nano-HPLC. Several peptides resulted to be fragments derived from salivary precursor proteins, possibly reflecting the protease activity in the periodontal pocket (see Table 1). In this study, however, the identification of the three putative alpha-defensins peaks (HNP_1–3_) (*m*/*z* = 3370.9, *m*/*z* = 3442.2, and *m*/*z* = 3486.5), detected in the MALDI-TOF spectra and also revealed by previous investigations [17,19] was complicated because precursors ions did not fragment by collision-induced dissociation in MS/MS mode, due to the presence of the three characteristic internal disulfide bonds inside defensins. Therefore, the authors validated a putative assignment by using a reducing agent and showing that the resulting MALDI-TOF mass spectra displayed a +6 Da shift only in the mass of three peaks, finding consistent with the reduction of three internal disulfide bonds. Other peptides and small proteins were also identified alternatively by SDS-PAGE followed by in-gel digestion and MS analyses using both nano liquid chromatography (LC)-ESI-MS/MS and MALDI-TOF/TOF MS. All the peptides and small proteins of this study are summarized in Table 1, while medium-size and larger proteins, also identified by the authors, are not listed in Table 1 because they’re outside of the scope of the present review.

Later on, the same group demonstrated that the combined use of MALDI-TOF MS with bioinformatics may allow the site-specific prediction of periodontal disease progression in a cohort of forty-one periodontal maintenance subjects [21]. Each individual and specific site were followed over 12 months, with clinical measurements taken both at baseline and then every three months, collecting GCF to be analyzed at each visit [21]. The authors compared the MALDI-TOF MS spectra of GCF of both healthy and diseased sites from patients with moderate to severe chronic periodontitis. The purpose of the study was to use these spectra to create models for disease diagnosis. In particular, to pursue this, they used a genetic algorithm to create a pattern analysis-based model to predict specifically sites which were undergoing attachment loss. Interestingly, the statistical algorithm showed that there was more similarity in the GCF profile between patients, than between sites with different severity of inflammation. However, even with these differences, MALDI profiles may be taken into account for the prediction of periodontal attachment loss at a site level with a very significant positive predictive value and, most importantly, before it can be measured by clinical examination. This valuable result can be achieved with one single test on each individual GCF sample. Remarkably, this was the first study which explored and preliminarily demonstrated the potential use of MALDI-TOF MS technology in the field of periodontal disease diagnostics.

Our group set up a standardized protocol for both GCF collection from healthy gingival tissue belonging to subjects diagnosed healthy by clinical examination and further GCF processing [22]. Pre-analytical and analytical variables expected to influence the GCF peptidome profiling were assessed by MALDI-TOF MS analysis. More specifically, in a comparative study, we explored how the robustness and reproducibility of MALDI-TOF MS profiles are influenced by the use of paper points vs. paper strips, by the conditions of centrifugal elution and by the utilization of PIC (Table 1). Moreover, the effects of both sinapinic acid composition and sample-to-matrix ratio in MALDI-TOF MS analysis were systematically estimated, with the aim of optimizing both the quality and robustness of signals in GCF MALDI-TOF spectra. Definitively, a simple and high throughput procedure was optimized to rapidly obtain reproducible peptidome profiles of GCF from healthy sites of clinically healthy subjects. Sample composition and dilution severely affected the peptidome profiles obtained in MALDI-TOF analysis, therefore in this study, the optimal elution volume was evaluated in the assessment of a standardized procedure. Among other findings, elution by TFA generated richer gingival fingerprints compared to those obtained by the use of acetic acid. PIC supplementation and centrifugation speed did not change GCF profiles. Matrix composition and matrix/sample volume ratio were also assessed to obtain robustness of the MS analysis ensuring CV less than 10% for peak area and signal-to-noise ratio. These results are of interest for standardizing both sample collection and handling methods for GCF peptidomic-based biomarker identification, although we are aware that the procedure we set up may have to be re-visited for clinical conditions differing from a healthy status (for instance, gingival inflammation or periodontal disease), in which it is known that the nature of the GCF changes dramatically.

As diagnostic peptide fingerprints may vary as a function of GCF collection, handling, and storage, our research group analyzed if storage conditions have an influence on the quality and the reproducibility of MALDI-TOF profiles for this biological fluid [23]. Indeed, our data strongly suggest that sample storage conditions indeed affect the GCF peptide pattern over time in a quite significant manner. Specifically, a procedure of immediate extraction of GCF from paper strips, followed by storage of the extracted material, generates a lower amount of variation in molecular profiles as compared to the extraction performed after the storage on paper strips. Indeed, significant spectral changes were observed for GCF samples stored at −20 °C directly on the paper strips for three months and then extracted, in comparison to samples extracted immediately and then stored for three months in the same conditions. Moreover, a significant decrease in the peak area of HNP-3, of S100-A8 and of both full-length S100-A9 and its truncated form was detected after 3 months even at −80 °C when storage was on paper strips. The artifacts found in the “paper-stored GCF” profile may not only influence the pattern-based biomarker discovery, but also make its use not suitable for in vitro diagnostic test targeting. So S100-A8 and S100-A9 are proposed as periodontal disease potential diagnostic biomarkers [49]. This study definitively shows that the signatures closest to those obtained with immediate elution and analysis were attained for the GCF samples eluted in TFA and then immediately stored at temperatures not higher than −80 °C for at most one month. The information gained from our findings on protein/patterns stability after storage may be useful in defining standardized protocols enabling optimal preservation of GCF samples.

Very recently, our group proposed a fast and easy to perform method for processing GCF before MS analysis, thus minimizing the risk of false positive detection due to degradation of particularly sensitive molecular species within GCF samples [24]. In this investigation, twenty subjects (ten healthy and ten suffering from gingivitis) were recruited in order to perform comparative proteomics profiling studies between healthy and gingivitis subjects (Table 1 and Table 2). So, we proposed a standardized methodology for GCF analysis by MALDI-TOF/TOF-MS in order to identify a characteristic peptide signature of gingivitis. Storage times were established of one and three months concerning GCF specimen to analyze, in order to better evaluate any degradation and changes in GCF peptidome patterns. Comparative analysis of normalized peak area from peptidome profiles showed a pattern of five *m*/*z* peaks (3371, 4136, 4964, 13,153, and 13,458) which discriminate the two groups in a statistically significant manner. Specifically, the normalized peak area for *m*/*z* 3371, 4136, and 13,458 was significantly higher in GCF from gingivitis subjects compared to GCF from the healthy group, while an inverse trend was observed for *m*/*z* = 4964 and for *m*/*z* = 13,153 (see Table 2). The peak with *m*/*z* = 3371 was identified as the HNP-2 belonging to the triplet of alpha-defensins. Curiously, no significant change was observed for HNP-1 and HNP-3 between healthy and gingivitis groups. The other four peptides were identified as the C-terminal fragment of alpha-1-antitrypsin (AAT), namely C-36 peptide (*m*/*z* = 4136), as the β-thymosin (*m*/*z* = 4964) and, finally, two different post-translational modifications (PTMs) of the full-length S100-A9 protein were found (*m*/*z* = 13153 and *m*/*z* = 13458) (see Table 1). The study also demonstrated the emerging role of MALDI-TOF MS in the high-throughput characterization of naturally occurring peptides from complex bodily fluid and its potential to directly identify constituents of the GCF peptidome and their proteoforms.

In a very recent study, Tang et al. analyzed GCF, saliva, and serum samples from 50 gender- and age-matched subjects suffering from chronic periodontitis (*n* = 17) and gingivitis (*n* = 17), and from healthy subjects (*n* = 16), in order to identify potential biomarkers of periodontal diseases by using MALDI-TOF MS [25]. GCF samples were collected by paper strips, immediately deposited into a tube containing PBS; supernatants were recovered after centrifugation and stored at −80 °C (Table 1). GCF, saliva and serum samples were fractionated using a weak cation exchange magnetic beads and analyzed immediately on a MALDI-TOF mass spectrometer or stored at −20 °C and analyzed within 24 h. In the comparison between chronic periodontitis and healthy groups, four GCF peptides were significantly higher in chronic periodontitis group compared with healthy (3434.4 Da, 4126.6 Da, 5407.7 Da, and 5416.0 Da) (see Table 2). Among these, only the peak at *m*/*z* 3434.4 was successfully identified to be derived from haptoglobin by nano-LC/ESI-MS/MS analysis and its upregulation in chronic periodontitis patients was confirmed also for saliva samples. The authors identify other two peptides derived from haptoglobin both in serum and saliva (3874.9 Da and 1147.1 Da respectively) with an increased expression level in chronic periodontitis and gingivitis patients, thus confirming the results obtained with GCF. One strength of this study relies on the possibility to obtain specific molecular signatures for a given bodily fluid and then to compare the potential biomarkers identified across different bodily fluids. However, an important limitation, as pointed out by the authors themselves, is the absence of any kind of normalization for protein concentration. Furthermore, samples stored for the same amount of time need to be compared in order to ensure the robustness of the results; indeed, as discussed above, the protein profile of a sample analyzed immediately by MS may not be comparable to that of the same sample stored at −20 °C and analyzed within 24 h [23,24].

On the basis of blind MALDI-TOF analysis presently used in clinical microbiology for conventional bacterial strain identification, Antezack and colleagues adapted this analytical approach to MALDI peptide profiles acquired from GCF, saliva and dental plaque with the aim to develop a rapid diagnostic test for periodontitis [26]. They compared the MALDI spectra to assess if a classification may be performed among individuals according to their periodontal status. In particular, the authors used the binary discriminant analysis method [65] to evaluate differences between the groups of periodontitis (*n* = 67) and healthy periodontal (*n* = 74) subjects on the basis of a discriminant peak list (Table 1). Based on the differentially expressed peaks from MALDI peptide fingerprints, a diagnostic decision tree for periodontitis was built for each type of sample. In the case of GCF, a pattern of nine peaks was chosen to set up a decision tree with a specificity of 98% and a sensitivity of 96%. In a blind experiment GCF data demonstrated the ability to segregate periodontitis patients from control individuals showing a specificity of 75.7% (±0.195) and a sensitivity of 79.6% (±0.188). Although the authors claim their study as the first “to demonstrate that MALDI-TOF MS differentiates periodontitis from healthy periodontium by blind identification of specific patterns in mass signals from protein profiles in saliva, GCF and dental plaque” [26], the investigation suffers several limitations, in particular the absence of preliminary experiments designed to assess the impact of short-term storage at 4 °C of the analyzed samples and the total absence of quantification of total protein concentration in the same samples. In fact, concerning the first limitation (impact of short-term storage at 4 °C) although data in the literature show the small molecules like uric acid or cortisol are stable when saliva was stored for at 4 °C for four weeks [66,67] this may absolutely not hold true for protein or peptide components in the same fluid [23,24]. Further studies are absolutely necessary in order to increase the robustness of the diagnostic approach.

### 2.3. ESI MS GCF Peptidome Profiling

Unlike the MALDI source which ionizes and sublimates the samples out of a crystalline, dry matrix by laser, the ESI source ionizes the analytes out of a solution and is therefore usually coupled to LC instruments [68]. In the ESI source, the solution containing peptides analytes is passed through a needle to which a high voltage (~2–5 kV) is applied [69]. This leads to the generation of a spray of small charged droplets. As the solvent evaporates, desolvation of peptide/protein-solvent droplets resulted in a multicharged ions formations. The ions are then accelerated into the mass analyzer for subsequent measurement of their mass-to-charge ratios and of ion intensity. A mathematical deconvolution performed by the software on the ESI spectrum allows the mass of the analyte to be determined with an accuracy of roughly 1/10,000 amu. The combined information of both the time of elution (obtained, when possible, by using protein standards) and precise mass value of the protein quite often allows non ambiguous identifications to be obtained. This platform may be used successfully for the analysis of small complex samples because it allows high sensitivity detection of different analytes at the same time.

The mass analyzers routinely used with ESI are quadrupole, linear ion trap, and Orbitrap, however we concisely describe only the last two analyzers, those used in the peptidomic investigations here reviewed. Briefly, in a linear ion trap, ions are confined in radial direction by a two-dimensional radio frequency field, and in axial direction by stopping potentials applied to end electrodes [70]. The trapped ions are manipulated employing direct current and radio frequency electric fields in a series of carefully timed events. They can work as stand-alone mass spectrometers or into hybrid configurations combined with high-resolution mass analyzers. A correlation between the concentration of a given peptide and the ionic current, which is measured by the ion trap mass spectrometer, is however possible only under very reproducible conditions. Indeed, as the ionic current is directly proportional to the protonation level of the peptide, it is necessary to standardize the treatment of the samples. The linear ion trap mass spectrometer has become popular because of its robustness, fast scan speed, sensitivity, user friendliness and relatively low cost. However, resolution, mass accuracy and dynamic range are still not comparable to other mass spectrometers such as Orbitrap instruments [71]. Orbitrap is a benchtop instrument that is cost effective, accessible, and applicable in hybrid architectures. The Orbitrap MS is composed of a barrel-like outer electrode and a spindle-like central electrode [72]. In this electric field, ions rotate around the central electrode while oscillating down the length of the electrode; the frequency of these harmonic ion oscillations, undergone by the orbitally trapped ions, is used for *m*/*z* measurement [72]. The main advantage of the Orbitrap analyzers are enhanced resolution (the highest available for a benchtop mass spectrometer mostly in hybrid architectures), high mass accuracy, high sensitivity, wide dynamic range, fast acquisition rates and multi-stage MS^(*n*)^ capabilities for tandem MS experiments [71,73]

Pisano and colleagues were the first group to analyze GCF by reverse-phase (RP)-HPLC coupled to ESI MS [27]. The authors analyzed the acidic-soluble protein content of GCF collected under physiological conditions. After the chromatographic separation, the eluate was directly introduced into an ion-trap mass spectrometer through ESI. Concerning peptides and small proteins, the HNPs 1–3 (alpha-defensins 1, 2, and 3) were the principal components detected, while only minor amounts of HNP-4, statherin, PB peptide, cystatin A were observed. Additionally, other unidentified components were detected with low mass values of 1067.4, 1109.4, and 1151.4 amu (probably corresponding to the same molecule showing a different degree of acetylation) and other peaks with mass values of 4135.0, 4936.4, 4964.0 and 4980.0 amu. This was also the first study to analytically demonstrate that GCF represents a physiological entity distinct from saliva. In fact, the authors did not detect the presence in chromatogram of the major proteins/peptides characteristic of human saliva such as acidic and basic proline-rich proteins. Although this approach does not allow for the detection of proteins that are not soluble in acidic solution, it contributed to the initial effort to discover the still under-represented GCF peptidome by an innovative MS-top down approach.

The same group (Inzitari et al.) demonstrated later, not only by HPLC-ESI-MS but also by immunohistochemical analysis, that gingival sulcus is the main source of Tβ4 in the oral cavity and that GFC contains high levels of Tβ4 and also of a second member of the same peptide family, namely Tβ10 [28]. Of note, these peptides play a key role in diverse cellular functions such as tissue development, migration, angiogenesis, and wound healing [28].

Very recently, Dassatti and colleagues also performed a pilot study analyzing the GCF proteomic profile by RP-HPLC coupled to ESI MS. GCF samples were collected during the first 15 post-partum days from 10 female patients with a clinical scenario of gingivitis and periodontitis (assessed by clinical periodontal parameters), and from the same patients after three months in order to evaluate the effects of a professional oral hygiene session and to analyze the associated variations of proteomic profile of GCF. A control group was created in order to compare the results with GCF samples from 10 not pregnant fertile women [29]. The analysis of clinical periodontal parameters at three months post-partum revealed the therapeutic support of the professional oral hygiene session performed during the first visit. Proteomic profiling of GCF, performed by RP-HPLC-ESI coupled to a hybrid ion trap-Orbitrap mass spectrometer, highlighted the presence of peptides playing a key role during the inflammatory process and exerting defense mechanisms. These peptides, were then identified by MS/MS experiments as α-defensins, thymosin-β4 with one of its fragments (21–44), thymosin-β10, fibrinopeptide A and its fragments (fragments 21–35 and 22–35) and fibrinopeptide B (Table 1). The levels of all the defensins (α-defensins 1–4) and of all the fibrinopeptides, were always higher during post-partum gingivitis and periodontitis, compared to the three months recall group and the control group (Table 2). The value of thymosin-β4 decreased in the group of patients with periodontitis, while it was significantly increased in the three months recall group. A similar trend was also observed for thymosin-β10 (Table 2). Otherwise, the expression level of thymosin β4 fragment (21–44) was higher during post-partum periodontitis compared to the three months recall group and the control group (Table 2). These findings demonstrate that pregnant women may be at an increased risk of pathological dental conditions and underscore the importance of a good oral hygiene procedures in helping to prevent problems with dental health. It is also interesting to associate the study of the expression level of peptides involved in the inflammatory process and in defense mechanisms with the variation of the clinical parameters normally used for the evaluation of the gingival health status. However, the paper lacks some important information about sample collection and processing.

## 3. The GCF Antimicrobial Peptidome

This section will focus in particular on the main findings concerning naturally occurring peptides, identified in GCF by profiling strategies performed without a previous proteolysis step propaedeutic to mass analysis. Considerations about their putative role and how their analysis might lead to the identification of novel biomarkers of periodontal health or disease will be discussed. As emerged from the literature reviewed in the previous sections, the top-down analysis of GCF peptides and their proteoforms (as they occurred in their intact form) poses several challenges for MS-based proteomics platforms. One of the main limitation arises in such cases from the short sequences of detected peptides, therefore the probability to find in their sequence basic aminoacidic residues is very low and as consequence those peptides may not be favorably charged for MS detection, as they are often singly charged and more difficult to efficiently fragment. Additionally, unlike bottom up approaches, the absence of Lys and Arg as non-tryptic peptides, hinders their detection. Furthermore, the conventional database search approach, which is normal routine in proteomics, is not suitable in this case. However these issues are extensively addressed also elsewhere [74,75]. Although definitive identification of naturally occurring peptides is particularly challenging, some MS instruments for example MALDI-TOF/TOF and ESI-ion trap or ESI-ion trap/Orbitrap provide not only a rapid screening of proteoforms [18,22,23,27], but also their sequencing and identifications [6,24,25,28,29]. In the light of the above considerations, it is worth highlighting important results from several groups; for example Ngo and colleagues detected and identified for the first time histone protein or peptide in GCF by MALDI-TOF/TOF (Table 1) [6]. Interestingly, histone fragments, have been shown to have antibacterial activity [76]. Other examples are the discovery of haptoglobin fragment in GCF by Tang et al. [25] (Table 1) as well as fibrinopeptide fragments by Dassatti et al. [29] (Table 1). It would possible to speculate that these fragments might play a more specific role in GCF compared to those played by their precursor protein. It is well known that haptoglobin is an acute-phase protein, which promotes anti-inflammation activities and could also act indirectly as an antioxidant and bacteriostatic agent [77]. Its expression increases in response to injury or infection as it contributes to the recovery of homeostasis after systemic or local infection [77]. Therefore, the authors hypothesized that the up-regulation of haptoglobin derived fragment levels in chronic periodontitis patients compared to healthy subjects (Table 2) may reflect the anti-inflammatory and reestablishment process of the periodontium. Fibrinogen and fibrinopeptides are involved in hemostasis, wound healing, inflammation, angiogenesis, vascularization after injury, and other biological functions [78]. So, the authors speculated that the increased levels in periodontal disease conditions of fibrinopeptide A and its fragments along with fibrinopeptide B (see Table 2) may be due to their involvement in the healing process and revascularization of the area affected by periodontal disease.

Another point worthy of note was the detection of Thymosin β4, together with its sulfoxide form, and of Thymosin β10 by Inzitari and colleagues [28] which demonstrated that gingival sulcus is the main source of Tβ4 in the oral cavity and that GFC is the main source of Tβ4 and Tβ10 [28]. Although several bottom-up approaches documented the presence of Thymosin β4, they failed to identify Thymosin β10 or the sulfoxide form of Thymosin β4 [14,32,39,42,45]. These peptides have multiple diverse cellular functions, including tissue development, migration, angiogenesis, and wound healing [79]. In particular, multifunctional roles are attributed to Tβ4 in protecting cells against damage by acting as antimicrobial, anti-inflammatory, and anti-apoptotic agent [80].

Our group detected two proteoforms of S100-A9 with *m*/*z* = 13,153 and 13,458 and the oxidized forms of S100-A8 [23,24]. S100-A9 together with S100 A8 belong to the damage-associated molecular pattern family members of danger signals that initiate and amplify local inflammation and innate immune responses [81]. S100-A9 is frequently associated with S100-A8 to form the S100-A8/S100-A9 heterodimer, known as calprotectin, with anti-microbial, chemotactic, pro-apoptotic and anti-proliferative activities [81]. Several studies shed light on the key role of calprotectin and its subunits in the pathobiology of a number of inflammatory conditions including periodontal diseases [49,81,82]. In addition the GCF calprotectin levels were positively correlated with clinical parameters of periodontitis [81]. Specifically, in our comparative study, we detected the acetylated form (*m*/*z* = 13153) and the acetylated and glutathionylated form of the S100-A9 (*m*/*z* = 13458) [24]. The first was found decreased in gingivitis patients compared to the healthy controls, while the latter was found increased [24]. It is common knowledge that GCF contains significant levels of reduced glutathione which is responsible for the local antioxidant capacity, typical of periodontally healthy subjects [83] and that glutathionylation of S100-A9 alters its ability to form complexes with S100-A8, to bind endothelial cells, and limits neutrophil migration in inflammatory lesions [84]. Based on these results, our group speculated that the glutathionylation in S100-A9 may result in a protective effect against oxidative process at the site of inflammation thus providing a coherent explanation to the observed reversed ratio between the *m*/*z* = 13,458 (both glutathionylated and acetylated) and the *m*/*z* = 13,153 (acetylated only) forms of S100-A9 peptide in gingivitis patients compared to healthy subjects [24].

Lundy and colleagues, in line with other transcriptomics and proteomics studies [4], found increased expression levels of S100-A8 in periodontitis and gingivitis sites compared to the healthy ones (Table 2) [16]. However, it is worth noting that, in some comparative proteomics studies, S100-A8 and S100-A9 are not differentially expressed, even if detected [32,33,34,41], suggesting various hypotheses as already reported elsewhere [23] and discussed in the next sections.

Among several AMPs detected by the MS profiling platforms, here described and summarized in Table 1 and Table 2, important families such as human alpha-defensins (HNPs 1–4) and human beta-defensins (hBD-1 and hBD-2) and also the cathelicidin antimicrobial LL-37 peptide were studied.

Concerning the alpha defensins (HNPs 1–4) the results of comparative studies between healthy and gingivitis groups or healthy and periodontitis groups (Table 2) showed that defensins, with the exclusion of one study [19], are generally increased in periodontal conditions (gingivitis and periodontitis, Table 2). Similarly to other AMPs, defensins exert their protective role against microbes entering the oral cavity resulting in effective control of infections. However, even with a broad bactericidal effect, HNPs have been demonstrated to be rather ineffective in vitro against various periodontal pathogens, at least at concentrations in which they are found in healthy gingival crevice [85]. Increased expression of α-defensins may induce tissue damage both directly, by their observed cytotoxic effects on cells [86], and indirectly, by competing with neutrophil elastase for the binding of α-1-antitrypsin (AAT), an inhibitor of neutrophil elastase [87]. Consequently, defensins may influence the balance between proteases and their inhibitors and increased defensins levels observed both in peptidomics studies (Table 2) [17,18,24,29] and also in proteomics investigations [34,38,40] may cause augmented proteolytic activity, thus contributing to the inflammation and the tissue damage observed in gingivitis. As shown in Table 2, in our studies we detected significantly increased levels of only HNP-2 in the gingivitis group while no significant change was observed for HNP-1 and HNP-3 between healthy and gingivitis groups [24]. Our results were partially in agreement with the findings of Dommisch et al. who observed increased levels of HNP-2 and, to a lesser extent, of HNP-1 (although both not statistically significant) in gingivitis sites in comparison to the healthy sites while no variation of HNP-3 was reported [17]. However, the general observed trend of increase of defensins in periodontal diseases was inverted in the study of Lundy and colleagues as they found that the defensins expressions were more abundant in a higher proportion of the healthy sites in comparison to the diseased (although the differences were not statistically significant) [19].

Interestingly, Diamond and colleagues detected in GCF not only the alfa defensins, but also the β -defensins hBD-1 and hBD-2, although hBD-1 was also found “in several smaller forms” suggesting extracellular proteolysis [18]. It is interesting to note that a higher amount of both hBD-2 and α-defensins, with little hBD-1, was observed in the subject with higher levels of inflammation (individual 1, Table 2). This observation is in line with increased levels of both hBD-2 and neutrophils in inflammation. β-defensins are small cationic peptides produced by gingival epithelium that are involved in the innate host defense against the bacterial challenge, which is continuously present in the oral cavity [88]. β-defensins play a key role in the awakening of the innate immune response to increased bacterial exposure in the gingival epithelium and exert combined antimicrobial, chemotactic and anti-inflammatory properties. These peptides contribute to the healing process of gingival wounds, regenerating the damaged epithelium by promoting the attachment and proliferation of fibroblasts on the diseased root surfaces [88].

Another relevant antimicrobial peptide detected by Dommisch and colleagues is the human cathelicidin LL-37, a peptide of 37 amino acid starting with two leucine residues which has a broad spectrum of antimicrobial activity [89]. It is stored as biologically inactive precursor in the secondary granules of neutrophils and it acquires its antimicrobial potency after proteolysis exerted by proteinase 3. LL-37 regulates inflammatory and immune responses, promotes angiogenesis and wound healing and, finally, also neutralizes lipopolysaccharides. The increased expression of this peptide in samples obtained from sites characterized by gingivitis compared to healthy periodontal sites (Table 2), together with the increased expression in the same diseased sites of HNP-1 and HNP-2, induced Dommisch and colleagues to speculate that LL-37 might be a good indicator of the antimicrobial capabilities of the gingival apparatus provided by neutrophils and that there might be an association between a parallel secretion of these AMPs due to the increased numbers of neutrophils in inflammation.

Our group identified for the first time the sequence of the peptide *m*/*z* = 4136 already detected in GCF in previous top down proteomics studies, but not structurally elucidated [27]. Despite technical issues, due not only to the complexity of GCF mixture but also to the high molecular weight of the precursor ion (*m*/*z* = 4136), a direct high-energy CID fragmentation of the selected peak allowed us to identify it as C-36 peptide corresponding to the residue fragment 383–418 of AAT protein [24] (Table 1). This peptide showed a statistically significant increase in gingivitis group compared to healthy group as previously described in the Section 2.2 [24] (Table 2). Interestingly, C-36 peptide deriving from AAT, exerts significant pro-inflammatory activity [90]. In fact, it was found expressed in human lung tissue as pro-inflammatory activator of human monocytes [91]. Moreover, the proteolytic cleavage of AAT at sites of inflammation may reverse the anti-inflammatory effect of AAT thus contributing to neutrophil recruitment and activation [92]. Considering that inflamed sites are populated by several bacterial species secreting proteolytic enzymes able to generate protein-breakdown products [93], it is tempting to speculate that the C-36 peptide, which was found increased in gingivitis patients as compared to healthy subjects [24], might be derived by AAT bacterial proteolytic breakdown. It should be stressed that a bottom-up LC MS/MS approach would have failed to address the levels of AAT endogenous fragment, due to the requirement for the proteomic mixture to be digested before it can be mass analyzed, whereas our top down MALDI-TOF platform allowed for the detection of increased levels of C-36 peptide in the GCF of gingivitis patients [24].

All these findings further demonstrate the utility of peptidomics profiling platforms for screening and detecting proteoforms, including products of specific proteolytic cleavages, which can have different effects and important biological roles on periodontal diseases processes. The ability to characterize these species as they occur in GCF is essential for understanding the mechanism of the pathobiological response to oral diseases.

## 4. Relevance and Challenge of Pre-Analytical and Analytical Variables

To date, the effects of different sampling protocols and the influence of both pre-analytical and analytical conditions on GCF proteomic profiling have not been extensively addressed in many of the proteomics and peptidomics investigations which analyzed this specific biological fluid. The inherent limitations of GCF, due to its tiny amount in healthy gums as well as its heterogeneity in periodontal diseases, pose many concerns especially in comparative studies, which require rigorous protocols for protein recovery and normalization of MS data. Other concerns arise from artefacts due to storage conditions. Furthermore, in many cases, investigations which are biased by inter-individual variability of GCF samples, were performed on too small size groups to guarantee sufficiently robust statistical analysis. Last but not least, the comparison of the results obtained among several research groups is not so straightforward and the strategy adopted (bottom-up or top-down) should also be taken into account.

### 4.1. Quantitation Issues

More than five decades of scientific literature have definitively demonstrated that different sampling methodologies, different sampling times and different processing protocols significantly affected both the quality and the quantity of GCF samples [94]. Up to date, only a few quantitative proteomics investigations have been performed with the aim to differentiate GCF protein profiles in periodontal health and disease [14,30,33,38,39,40,44]. In MALDI-TOF MS, but also in other MS platforms, the ionization is influenced not only by the analyte concentration, but also by its primary structure (in fact, the presence of one or more basic residues in a peptide/protein promotes the ionization). Therefore, in the MS investigations which analyze peptides without an hydrolysis preceding step, MS spectra and MS tandem spectra could result poor and in such cases the spectra interpretation may result challenging in comparison to those obtained by bottom-up procedures in which the presence of tryptic peptides facilitates the ionization and the fragmentation step in MS/MS analysis. The lack in some peptides of a positive charge on the N-terminal residue due to PTMs (acetylation/pyroglutamylation) and the simultaneous absence of basic residues such as Lys, Arg, or His hinder the detection of these peptides [74,75].

Concerning peptidomics studies, the quest for quantitative approach is stringently desirable in order to make published data comparable between each other. The lack of data uniformity is due to several factors, including the difficulty in accurately measuring the small volumes of GCF obtained (especially for healthy sites) and the differences both in collection methodology and processing protocols, as pointed out in Table 1. An additional problem, which complicates the analysis and standardization issues in GCF analysis, is the amount of volume used to elute the sample collected on paper strips or on paper points. In particular, this is an important issue in the case of SELDI and MALDI based investigations (Table 1). In fact, it is well accepted that MALDI-TOF MS spectra of complex bodily fluids are heavily influenced by the extent of sample dilution [63,95,96]. To address this issue in our investigations, experiments at different elution volumes have been performed and both the number of peaks and total peak area in MALDI-TOF MS spectra have been assessed [22]. Indeed, the observation that more concentrated solutions showed both a lower number of peaks and a lower total peak area in comparison to more diluted samples was the most evident and expected behavior; not surprisingly, this was consistent with the MALDI ionization process. Furthermore, since GCF is a complex biological sample for the presence of endogenous peptides and proteins of different structures and different concentrations, the occurrence of ion suppression effects may also take place [97,98].

The effect of heterogeneity of both sample/matrix and ion signals determines poor MALDI data reproducibility, making this kind of analysis unsuitable for quantitative purposes [62]. In order to make MS profiling analysis more robust the use of internal standard and/or a better assessment of peak area normalization are required. Several studies have shown that with the appropriate internal standard, linear calibration curves can be generated providing a correct quantification especially for small molecules and peptides, even in complex biological matrices [62,99,100,101]. The use of internal standard can be avoided when a linear correlation between analyte concentration and ion counts can be demonstrated [102]. A possible alternative to the utilization of an internal standard is the use of Ionic-Liquid Matrices [103].

### 4.2. PIC

The presence in the GCF of active proteases, of both human and bacterial origin might constitute serious drawbacks making GCF peptidomics analysis very challenging. As in saliva and in other bodily fluids, the degradation and/or protease activity in GCF should be taken in great account. In light of possible protease activity, in such peptidomics studies after sample collection the use of protease inhibitors or of other precautions to prevent protease activity might be highly recommended. Concerning the solutions or buffer used to elute GCF collected on paper strips, in our experience, the use of acidic aqueous solutions is preferable to neutral or basic aqueous/organic ones, because acidic elution quenches protease activity, preserving proteins from potential protease degradations [27,104]. It is interesting to underline that the use of acidic buffer in peptidomics investigations might turn useful also for other reasons. In fact, high molecular weight proteins such as mucins, lactoferrin, α-amylases, and myeloperoxidases, found to be present in GCF [14,30,31,33,34,36,37,38,40,41,42,43,44,45,46,47] and which could hinder the detection of peptidic components [105], are insoluble in acidic conditions [27]. Studies on peptidome stability of GCF samples with PIC supplementation at neutral or basic pH are not yet reported in literature. Many research groups make use of acidic buffer to elute or dilute GCF immediately after the collection [6,17,18,20,21,22,23,24,27,28] and, as indicated in Table 1, for all of the investigations reviewed, the use [22,23,24] or not [6,16,17,18,19,20,21,25,26,27,28,29] of PIC is shown.

The levels of the peptides present in bodily fluids might reflect the activity of protease(s) generating them, which could in turn be influenced by various biological events. Thus, the amount of certain peptides can be used as indirect sensors of the biological state of an individual and, in the end, could provide invaluable information for clinical diagnoses [57]. Among the peptidomics studies reviewed here, many investigations have shown in GCF the presence of peptide fragments derived by proteolytic cleavage from larger proteins such as Fibrinopeptide A fragments (21–35) and (22–35) and Fibrinopeptide B [29], haptoglobin derived peptide [25], Thymosin β-4 fragment (21–44) [29], hBD-1 derived peptides [18] and peptides fragments derived from salivary and serum precursor proteins [6], C36 peptide AAT [24]. Many of these peptides, arising from precursor proteins after exquisitely specific proteolytic cleavages, could exert significant biological activity against various kinds of microorganisms as already outlined in previous section. However, these findings currently remain only mere hypotheses and targeted experiments are required in order to support these possible speculations.

Careful attention should be used in the interpretation of the results of all the studies in which neither protease inhibitors nor other precautions to prevent protease activity were used during sample collection, as the presence of fragments identified as putative biomarkers of pathology may simply be due to the action of uninhibited proteases.

Ultimately, the presence of glutathione in stored GCF determines glutathionylation patterns of such protein target [23] lowering the concentration of the protein as demonstrated in our previous work [23]. The above considerations, together with the degradation patterns observed during storage in various conditions [23,24], underline the requirement to better asses the concentration and storage issues (see below) not only in top down but also in bottom up approaches in order to make the results comparable among each other for a consistency in data interpretation.

### 4.3. Storage

Among the various pre-analytical issues that deeply affect the peptide profiles of GCF, the storage conditions may strongly compromise the correct interpretation of data analysis if not properly assessed. To address this issue, our group studied the stability of GCF MALDI-TOF profiles after sample collection under different storage conditions [23,24]. Specifically, the storage of GCF immediately extracted from paper strips was found to generate less variations in molecular profiles compared to those obtained when the extraction is performed after the storage. In fact, significant spectral changes were detected for those samples stored at −20 °C directly on the paper strips and extracted after three months, in comparison to the freshly extracted control [23]. Still, even in the case of GCF samples immediately extracted from paper strips, a significant decrease in the peak area of HNP-3, S100-A8, full-length S100-A9, and its truncated form were detected after three months of storage, even at a temperature as low as −80 °C [23].

Based on the above findings, we conclude that the storage conditions may strongly compromise the correct data interpretation, since alterations of “stored GCF” profile may influence the pattern-based biomarker discovery. As a consequence, the samples to be compared (healthy versus periodontal diseases) should be stored for the same amount of time in order to minimize the chance that the results of the study may be biased by potential artifacts.

### 4.4. The Inter-Individual Variability

Considering the intrinsic inter-individual variability of GCF samples, large cohorts are necessary in order to compare the proteomes in the presence or absence of periodontitis. As reported in Table 2, only few case-control reports describing differences between healthy and gingivitis [24] or healthy and periodontitis subjects [19,25] or sites [16,17,29] were reported; moreover only one experimental model of feasibility blinded test [26] emerged (Table 2).

With the exception of the studies by Tang and colleagues (*n* = 50) [25] and by Antezack et al. (*n* = 141) [26], a limitation of the reports summarized in Table 2 is the small sample size.

It is quite conceivable that the subset of potential biomarkers identified in a small sample size might not be confirmed on a larger cohort of patients. Conversely, the use of larger sample sizes should provide increased statistical power allowing the identification of additional biomarkers. Investigations on larger cohorts could provide a more accurate qualitative interpretation of peptide signatures, for well-defined clinical applications.

## 5. Bioinformatics Approaches

It should be considered that the majority of data in spectral libraries are built from in-silico enzymatic digestion of proteins, limiting the analysis only to those peptides derived from expected enzyme cleavage sites and limiting the number of PTMs considered; for this reason the datasets are difficult to leverage for peptidomics [74]. Moreover, the limited length of the aminoacidic sequences makes peptidomics spectral data analysis and interpretation more challenging in comparison to conventional bottom-up proteomics. As already underlined in Section 3, the studies here reported relate to ‘non-tryptic’ peptides which generate MS/MS patterns less informative in comparison to the ‘tryptic digested peptides’. In fact, due to their intrinsic nature, the absence of basic Lysine or Arginine at the C-terminal sequence might hurdle the fragmentation process and, as a consequence, generation of poor MS/MS spectra could be observed. In line with these observations, the same bioinformatics strategies used for bottom-up approaches do not perform efficiently when less predictive and informative MS/MS patterns are obtained from endogenous peptides [75]. It is important to highlight that, unlike bottom-up proteomics, software solutions for peptidome characterization are not fully developed and data analysis often requires laborious manual interpretation and rigorous validation especially when the identification is based on a single peptide [74,75]. *De novo* sequencing algorithms assisted by classical database search programs for sensitive and accurate peptide identification are also currently available [106,107]. *De novo* sequence assignments (when manually performed) often require skilled and experienced personnel and, besides fragmentation patterns, further acquired confirmations for identification can possibly be the assessment of ion intensities and in the case of LC-ESI experiments, evaluation of accurate retention times [27]. The use of software for predicted fragmentations is one of most cost-effective way to validate the identification [108]. For example, concerning both MALDI-TOF/TOF and ESI experiments, when the automated software identify with low confidence endogenous peptide and their fragments, in order to overcame these difficulties and to reach high confidence identification/validation, the experimental mass value of the peptide derived from unspecific cleavage of precursor protein/endogenous peptide can be compared with average theoretical mass values using the FindPept or PeptideMass software (available at http://www.expasy.org/tools) and the experimental MS/MS spectrum can be compared to the MS/MS spectrum generated from the Protein Prospector (http://prospector.ucsf.edu/). Furthermore, several tools have been developed for peptide mapping such as iPiG, EnzymePredictor, PatternLab, Peptigram, UStags, PepNovo, and MS-Tag [74].

## 6. Conclusions

Peptidomics research represents one of the most interesting and challenging area of proteomics. GCF peptidome could be a goldmine for the discovery of novel biomarkers of periodontal diseases. While many proteomics-based bottom-up approaches have been pursued, resulting in a burgeoning of proteins occurring in GCF, still these approaches are not adequate enough to completely disclose all the proteoforms present in the sample. On the other hand, peptidomics investigations, mainly based on profiling (top-down) strategies, which are well suited for proteoforms detection, are very few and therefore, up to date, the picture of naturally occurring peptides and their role in GCF is still incomplete. In this scenario, the main limitations are due to the lack of standardization of pre-analytical and analytical variables affecting the different processing steps from the sample collection, to the potential artefacts coming from storage of GCF and from the protein concentration normalization and, finally, to the robustness of MS analysis. So, differences derived from heterogeneity in collection, handling, and storage make the results difficult to compare and to establish their correct interpretation due to their inconsistency. Controversies arising from defensins and calgranulins are clear examples of these data misinterpretations.

With the appropriate standardization, MS profiling strategies will allow to harvest intact peptide signatures from GCF which could help to delineate the subtle boundaries existing among the different stages of gingival inflammation. Obviously, each single biomarker forming the signature can be validated by more traditional biochemical approaches like, for instance, Western blot analysis or ELISA assays, which both present an advantage in terms of exquisite specificity of purposely developed monoclonal antibodies raised against the peptide identified by MS. This will open the possibility to recognize a specific phenotypic pattern for the early diagnosis and the progression to periodontal diseases. Moreover, an increased coverage of both GCF peptidome and proteome might assist the comprehension of molecular mechanisms underpinning the pathogenesis of periodontitis. Although the focus of this review is on periodontal diseases, in recent years it was proposed that GCF and saliva should be kept in consideration also in “the wider contexts of oral and systemic health” [109]. Indeed, it has been reported that saliva has shown potential for the development of diagnostic assays for systemic pathologies such as acquired immunodeficiencies, diabetes mellitus, Sjögren syndrome, cerebrovascular/cardiovascular diseases, systemic sclerosis, and even for cancers like breast cancer and, not surprisingly, more localized tumors such as tumors of oral cavity, salivary gland tumor larynx carcinoma, and head and neck carcinoma ([4] and references therein). Considering that GCF has a different origin compared to saliva (it is not secreted from the salivary glands) it is not surprising that these two biofluids do not have a perfect overlap as diagnostic predictors in systemic health, however it has been reported that periodontal diseases (focus of this review) and the consequent periodontal inflammation can have an effect on the progression of important systemic disorders, such as cardiovascular and cerebrovascular diseases [94]. In light of these considerations, MS profiling strategies will allow to harvest intact peptide signatures from GCF which can be used for diagnostics and prognostics assays for important systemic disorders, such as cardiovascular and cerebrovascular diseases.

## Figures and Tables

**Table 1:** Gingival crevicular fluid (GCF) peptidomics profiling studies.

Ref.	Collection Devices/Elution Buffer/ Pre-Processing	Storage Conditions	Protein Quantitation	MS Normalization	MS-Approach	Naturally Occurring Peptides &Small Proteins Detected: Main Results
Lundy et al. [16]	Paper strips/sodium phosphate containing NaCl; NO PIC; RP-HPLC.	−70 °C, time not specified.	Yes	Not required ^(a)^	N-terminal aminoacid sequencing and LC-MS	S100-A8
Diamond et al. [18]	Periopaper strips/5% acetic acid; NO PIC; ProteinChip.	Not specified.	No	No	SELDI-TOF	α-defensins 1–3, hBD-1 derived peptides, hBD-2
Dommisch et al. [17]	Periopaper strips/5% acetic acid; NO PIC; ProteinChip.	Not specified.	Yes ^(b)^	No	SELDI-TOF	α-defensins 1–3, Cathelicidin antimicrobial peptide LL-37
Lundy et al. [19]	Periopaper strips/sodium phosphate containing NaCl; NO PIC; Unfractionated.	−80 °C, time not specified.	Yes	No	MALDI-TOF	α-defensins 1–3
Wen et al. [20]	Paper points/2.5% TFA; NO PIC; Unfractionated.	−80 °C, time not specified.	No	Yes	MALDI-TOF	*m*/*z* = 1660.2, 1783.0, 2912.5, 4178.6, 5064.9, 6108.9
Ngo et al. [6]	Glass microcapillary tubes/water or 0.1% TFA for HPLC fractionation; NO PIC; ZipTips or RP-HPLC.	−70 °C, time not specified.	No	No	MALDI-TOF/TOF	α-defensins 1–3, Peptides fragments from: Peptide salivary low MW, Proline-rich phosphoprotein, Beta globin, Peptide PA saliva, (Peptide PB saliva, Peptide PC saliva, H2A histone family, Ig light chain variable region, Glyceraldehyde-3-phosphate, Fibroblast growth factor, Albumin, Collagen, Ig heavy chain, Thioredoxin, Statherin, Hemoglobin alpha 1, Albumin Dbox binding protein, Collagen R-1 type III, Metallopeptidase
Ngo et al. [21]	Glass microcapillary tubes/GCF (0.2–1.5 µL) was dispensed with 2.5% TFA; NO PIC; ZipTips.	−70 °C, time not specified.	No	Yes	MALDI-TOF	Attachment loss sites vs. stable sites (*m*/*z*): 2023.50, 4042.80, 4490.30, 4525.30, 5232.30, 5502.20, 6890.10, 10939.00, 12833.00, 14008.00
Preianò et al. [22]	Paper points, Paper strips/2.5% TFA or 5% acetic acid; PIC and NO PIC; Unfractionated.	GCF was immediately analyzed by MS.	Yes	Yes	MALDI-TOF/TOF	α-defensins 1–4, Thymosin β4
Preianò et al. [23]	Paper strips/2.5%TFA; PIC and NO PIC; Unfractionated.	−20 °C/1 or 3 months, −80 °C/1 or 3 months and immediately analyzed	Yes	Yes	MALDI-TOF/TOF	α-defensins 1–4, hBD-2, Thymosin β4, S100-A8 and its oxidized forms, S100-A9 and its isoforms, Lysozyme C
Preianò et al. [24]	Paper strips/2.5%TFA; PIC and NO PIC, Unfractionated.	−20 °C/1 month, −80 °C/1 month and immediately analyzed.	Yes	Yes	MALDI-TOF/TOF	α-defensins 1–3, hBD-2, C-36 peptide of AAT, Thymosin β4, Thymosin β10, S100-A8 and its oxidized forms, S100-A9 and its isoforms, Lysozyme C
Tang et al. [25]	Paper strips/phosphate-buffered saline; NO PIC; Weak cation exchange magnetic beads.	−80 °C, time not specified ^(c)^.	No	Yes	MALDI-TOF	Haptoglobin derived peptide and other unidentified peptides: *m*/*z* = 4126.6, 5407.7, 5416.0
Antezack et al. [26]	Paper points/HPLC-grade water; NO PIC; Unfractionated.	+4 °C and analyzed within 24 to 48 h.	No	Yes	MALDI-TOF	*m*/*z* = 3775, 4235, 4944, 5296, 5728, 5893, 10586, 11324, 11,359 and 11447
Pisano et al. [27]	Paper cones/0.2% TFA aqueous solution; NO PIC; RP-HPLC.	−80 °C, time not specified.	No	Yes	ESI-Ion Trap	α-defensins 1–4, Statherin, Peptide PB, Cystatin A
Inzitari et al. [28]	Paper cones/0.2% TFA aqueous solution; NO PIC; RP-HPLC.	Storage conditions not specified.	Yes	Yes	ESI-Ion Trap	Thymosin β4, Thymosin β4 sulfoxide, Thymosin β10
Dassatti et al. [29]	Paper cones/Buffer not specified; Use of PIC not specified; RP-HPLC.	−80 °C, time not specified.	No	Yes	ESI-Ion Trap/ Orbitrap	α-defensins 1–4, Thymosin β4, Thymosin β4 fragment (21–44), Thymosin β10, Fibrinopeptide A, Fibrinopeptide A fragments (21–35) and (22–35), Fibrinopeptide B

^(a)^ Peak normalization is not required as it is a targeted study. ^(b)^ Protein quantitation: samples from healthy periodontal sites served as internal control (baseline) for quantitative analysis. ^(c)^ After storage at −80 °C, GCF samples were fractioned using a weak cation exchange magnetic bead kit and then immediately analyzed on MALDI-TOF or stored at −20 °C and analyzed within 24 h.

**Table 2:** Naturally occurring peptides found in gingival crevicular fluid (GCF) from different study groups. Expression levels are shown as increased (↑) or decreased (↓), as determined by the referred investigation in relation to the appropriate control group. H = Healthy, G = Gingivitis, P = periodontitis, CP = Chronic periodontitis.

Naturally Occurring Peptides	Methods	Study Groups and Number of Subjects (*n*)	Study Groups and Protein Expression Level	Ref.
C-36 AAT	MALDI-TOF	H (10) vs. G (10)	↓H/↑G *	Preianò et al. [24]
Cathelicidin antimicrobial peptide LL-37	SELDI-TOF	H sites (*n* = 8) and G sites (*n* = 8) in subjects with good general health (4).	↓H sites/↑G sites in H *	Dommisch et al. [17]
Fibrinopeptide A	ESI-Ion Trap/Orbitrap	G and P sites in women after pregnancy (10) and not pregnant women as H controls (10).	↓H/↑P sites * ↓H/↑G sites *	Dassatti et al. [29]
Fibrinopeptide A fragment (21–35)	ESI-Ion Trap/Orbitrap	G and P sites in women after pregnancy (10) and not pregnant women as H controls (10).	↓H/↑P sites * ↓H/↑G sites *	Dassatti et al. [29]
Fibrinopeptide A fragment (22–35)	ESI-Ion Trap/Orbitrap	G and P sites in women after pregnancy (10) and not pregnant women as H controls (10).	↓H/↑P sites * ↓H/↑G sites *	Dassatti et al. [29]
Fibrinopeptide B	ESI-Ion Trap/Orbitrap	G and P sites in women after pregnancy (10) and not pregnant women as H controls (10).	↓H/↑P sites * ↓H/↑G sites *	Dassatti et al. [29]
Haptoglobin derived peptide	MALDI-TOF	CP (17) and H (16).	↓H/↑CP *	Tang et al. [25]
hBD-1	SELDI-TOF	Patients with mild to moderate gingival inflammation (2).	↑mild gingival inflammation/↓moderate gingival inflammation	Diamond et al. [18]
hBD-2	SELDI-TOF	Patients with mild to moderate gingival inflammation (2).	↓mild gingival inflammation/↑moderate gingival inflammation	Diamond et al. [18]
HNP-1	SELDI-TOF	Patients with mild to moderate gingival inflammation (2).	↓mild gingival inflammation/↑moderate gingival inflammation	Diamond et al. [18]
	SELDI-TOF	H sites (*n* = 8) and G sites (*n* = 8) in subjects with good general health (4).	↓H sites/↑G sites in H	Dommisch et al. [17]
	MALDI-TOF	P(11) and H (12)	↑H/↓P	Lundy et al. [19]
	ESI-Ion Trap/Orbitrap	G and P sites in women after pregnancy (10) and not pregnant women as H controls (10).	↓H/↑P sites * ↓H/↑G sites *	Dassatti et al. [29]
HNP-2	SELDI-TOF	Patients with mild to moderate gingival inflammation (2)	↓mild gingival inflammation/↑moderate gingival inflammation	Diamond et al. [18]
	SELDI-TOF	H sites (*n* = 8) and G sites (*n* = 8) in subjects with good general health (4)	↓H sites/↑G sites in H	Dommisch et al. [17]
	MALDI-TOF	P (11) and H (12)	↑H/↓P	Lundy et al. [19]
	MALDI-TOF	H (10) vs. G (10)	↓H/↑G *	Preianò et al. [24]
	ESI-Ion Trap/Orbitrap	G and P sites in women after pregnancy (10) and not pregnant women as H controls (10).	↓H/↑P sites * ↓H/↑G sites *	Dassatti et al. [29]
HNP-3	SELDI-TOF	Patients with mild to moderate gingival inflammation (2).	↓mild gingival inflammation/↑moderate gingival inflammation	Diamond et al. [18]
	MALDI-TOF	P (11) and H (12)	↑H/↓P	Lundy et al. [19]
	ESI-Ion Trap/Orbitrap	G and P sites in women after pregnancy (10) and not pregnant women as H controls (10).	↓H/↑P sites * ↓H/↑G sites *	Dassatti et al. [29]
HNP-4	ESI-Ion Trap/Orbitrap	G and P sites in women after pregnancy (10) and not pregnant women as H controls (10).	↓H/↑P sites ↓H/↑G sites	Dassatti et al. [29]
S100-A8	N-terminal amino acid sequencing and LC-MS	H, G and P sites in patients with P (15) and H sites in H subjects (5).	↓H/↑G sites in P * ↓H/↑P sites in P * ↓H sites in P/↑G sites in P * ↓H sites in P/↑P sites in P *	Lundy et al. [16]
S100-A9	MALDI-TOF	H (10) vs. G (10)	↑H/↓G *	Preianò et al. [24]
S100-A9 Glutathionylated	MALDI-TOF	H (10) vs. G (10)	↓H/↑G *	Preianò et al. [24]
Thymosin β-4	MALDI-TOF	H (10) vs. G (10)	↑H/↓G *	Preianò et al. [24]
	ESI-Ion Trap/Orbitrap	G and P sites in women after pregnancy (10) and not pregnant women as H controls (10).	↑H/↓P sites * ↑H/↓G sites *	Dassatti et al. [29]
Thymosin β-4 fragment (21–44)	ESI-Ion Trap/Orbitrap	G and P sites in women after pregnancy (10) and not pregnant women as H controls (10).	↓H/↑P sites * ↓H/↑G sites *	Dassatti et al. [29]
Thymosin β-10	ESI-Ion Trap/Orbitrap	G and P sites in women after pregnancy (10) and not pregnant women as H controls (10).	↑H/↓P sites * ↑H/↓G sites *	Dassatti et al. [29]

* Statistically significant difference.

## References

[1] Tonetti M.S., Jepsen S., Jin L., Otomo-Corgel J. (2017). Impact of the global burden of periodontal diseases on health, nutrition and wellbeing of mankind: A call for global action. J. Clin. Periodontol..

[2] Tonetti M.S., Greenwell H., Kornman K.S. (2018). Staging and Grading of Periodontitis: Framework and Proposal of a New Classification and Case Definition. J. Periodontol..

[3] Tsuchida S., Satoh M., Sogawa K., Kawashima Y., Kado S., Ishige T., Beppu M., Sawai S., Nishimura M., Kodera Y. (2014). Application of Proteomic Technologies to Discover and Identify Biomarkers for Periodontal Diseases in Gingival Crevicular Fluid: A review. Proteom. Clin. Appl..

[4] Trindade F., Oppenheim F.G., Helmerhorst E.J., Amado F., Gomes P.S., Vitorino R. (2014). Uncovering the Molecular Networks in Periodontitis. Proteom. Clin. Appl..

[5] Page R.C., Schroeder H.E. (1976). Pathogenesis of Inflammatory Periodontal Disease. A Summary of Current Work. Lab. Investig. J. Tech. Methods Pathol..

[6] Ngo L.H., Veith P.D., Chen Y.Y., Chen D., Darby I.B., Reynolds E.C. (2010). Mass Spectrometric Analyses of Peptides and Proteins in Human Gingival Crevicular Fluid. J. Proteome Res..

[7] Toczewska J., Konopka T., Zalewska A., Maciejczyk M. (2020). Nitrosative Stress Biomarkers in the Non-Stimulated and Stimulated Saliva, as well as Gingival Crevicular Fluid of Patients with Periodontitis: Review and Clinical Study. Antioxidants.

[8] Khurshid Z., Mali M., Naseem M., Najeeb S., Zafar M.S. (2017). Human Gingival Crevicular Fluids (GCF) Proteomics: An Overview. Dent. J..

[9] Barros S.P., Williams R., Offenbacher S., Morelli T. (2016). Gingival Crevicular as a Source of Biomarkers for Periodontitis. Periodontology 2000.

[10] Delima A.J., Van Dyke T.E. (2003). Origin and Function of the Cellular Components in Gingival Crevice Fluid. Periodontology 2000.

[11] Subbarao K.C., Nattuthurai G.S., Sundararajan S.K., Sujith I., Joseph J., Syedshah Y.P. (2019). Gingival Crevicular Fluid: An Overview. J. Pharm. Bioallied Sci..

[12] Majeed Z.N., Philip K., Alabsi A.M., Pushparajan S., Swaminathan D. (2016). Identification of Gingival Crevicular Fluid Sampling, Analytical Methods, and Oral Biomarkers for the Diagnosis and Monitoring of Periodontal Diseases: A Systematic Review. Dis. Markers.

[13] Griffiths G.S. (2003). Formation, Collection and Significance of Gingival Crevice Fluid. Periodontology 2000.

[14] Silva-Boghossian C.M., Colombo A.P., Tanaka M., Rayo C., Xiao Y., Siqueira W.L. (2013). Quantitative Proteomic Analysis of Gingival Crevicular Fluid in Different Periodontal Conditions. PLoS ONE.

[15] Bostanci N., Bao K. (2017). Contribution of Proteomics to Our Understanding of Periodontal Inflammation. Proteomics.

[16] Lundy F.T., Chalk R., Lamey P.J., Shaw C., Linden G.J. (2001). Quantitative Analysis of MRP-8 in Gingival Crevicular Fluid in Periodontal Health and Disease Using Microbore HPLC. J. Clin. Periodontol..

[17] Dommisch H., Vorderwülbecke S., Eberhard J., Steglich M., Jepsen S. (2009). SELDI-TOF-MS of Gingival Crevicular Fluid-A Methodological Approach. Arch. Oral Biol..

[18] Diamond D.L., Kimball J.R., Krisanaprakornkit S., Ganz T., Dale B.A. (2001). Detection of Beta-Defensins Secreted by Human Oral Epithelial Cells. J. Immunol. Methods.

[19] Lundy F.T., Orr D.F., Shaw C., Lamey P.J., Linden G.J. (2005). Detection of Individual Human Neutrophil Alpha-Defensins (Human Neutrophil Peptides 1, 2 and 3) in Unfractionated Gingival Crevicular Fluid-A MALDI-MS Approach. Mol. Immunol..

[20] Wen X., Gu Y., Chen F. (2016). Gingival Crevicular Fluid as a Novel Potential Source of Biomarkers Distinguishes Pubertal from Post-Pubertal Subjects. Diagnostics.

[21] Ngo L.H., Darby I.B., Veith P.D., Locke A.G., Reynolds E.C. (2013). Mass Spectrometric Analysis of Gingival Crevicular Fluid Biomarkers Can Predict Periodontal Disease Progression. J. Periodontal Res..

[22] Preianò M., Falcone D., Maggisano G., Montalcini T., Navarra M., Paduano S., Savino R., Terracciano R. (2014). Assessment of Pre-Analytical and Analytical Variables Affecting Peptidome Profiling of Gingival Crevicular Fluid by MALDI-TOF Mass Spectrometry. Clin. Chim. Acta.

[23] Preianò M., Maggisano G., Lombardo N., Montalcini T., Paduano S., Pelaia G., Savino R., Terracciano R. (2016). Influence of Storage Conditions on MALDI-TOF MS Profiling of Gingival Crevicular Fluid: Implications on the Role of S100A8 and S100A9 for Clinical and Proteomic Based Diagnostic Investigations. Proteomics.

[24] Preianò M., Maggisano G., Murfuni M.S., Villella C., Pelaia C., Montalcini T., Lombardo N., Pelaia G., Savino R., Terracciano R. (2018). An Analytical Method for Assessing Optimal Storage Conditions of Gingival Crevicular Fluid and Disclosing a Peptide Biomarker Signature of Gingivitis by MALDI-TOF MS. Proteom. Clin. Appl..

[25] Tang H., Yuan C., Ma Z., Zhu C., Tong P., Gallagher J.E., Sun X., Zheng S. (2019). The Potentiality of Salivary Peptide Biomarkers for Screening Patients with Periodontal Diseases by Mass Spectrometry. Clin. Chim. Acta.

[26] Antezack A., Chaudet H., Tissot-Dupont H., Brouqui P., Monnet-Corti V. (2020). Rapid Diagnosis of Periodontitis, a Feasibility Study Using MALDI-TOF Mass Spectrometry. PLoS ONE.

[27] Pisano E., Cabras T., Montaldo C., Piras V., Inzitari R., Olmi C., Castagnola M., Messana I. (2005). Peptides of Human Gingival Crevicular Fluid Determined by HPLC-ESI-MS. Eur. J. Oral. Sci..

[28] Inzitari R., Cabras T., Pisano E., Fanali C., Manconi B., Scarano E., Fiorita A., Paludetti G., Manni A., Nemolato S. (2009). HPLC-ESI-MS analysis of oral human fluids reveals that gingival crevicular fluid is the main source of oral thymosins beta (4) and beta (10). J. Sep. Sci..

[29] Dassatti L., Manicone P.F., Iavarone F., Stefanelli N., Nicoletti F., Lazzareschi I., Luciano R.P.M., Castagnola M., D’Addona A. (2019). Proteomic Evaluation of GCF in the Development of Pregnancy Related Periodontal Disease: A Pilot Clinical Study. Eur. Rev. Med. Pharmacol. Sci..

[30] Grant M.M., Creese A.J., Barr G., Ling M.R., Scott A.E., Matthews J.B., Griffiths H.R., Cooper H.J., Chapple I.L. (2010). Proteomic Analysis of a Noninvasive Human Model of Acute Inflammation and Its Resolution: The Twenty-One Day Gingivitis Model. J. Proteome Res..

[31] Kido J., Bando M., Hiroshima Y., Iwasaka H., Yamada K., Ohgami N., Nambu T., Kataoka M., Yamamoto T., Shinohara Y. (2012). Analysis of Proteins in Human Gingival Crevicular Fluid by Mass Spectrometry. J. Periodontal Res..

[32] Tsuchida S., Satoh M., Umemura H., Sogawa K., Kawashima Y., Kado S., Sawai S., Nishimura M., Kodera Y., Matsushita K. (2012). Proteomic Analysis of Gingival Crevicular Fluid for Discovery of Novel Periodontal Disease Markers. Proteomics.

[33] Tsuchida S., Satoh M., Kawashima Y., Sogawa K., Kado S., Sawai S., Nishimura M., Ogita M., Takeuchi Y., Kobyashi H. (2013). Application of Quantitative Proteomic Analysis Using Tandem Mass Tags for Discovery and Identification of Novel Biomarkers in Periodontal Disease. Proteomics.

[34] Baliban R.C., Sakellari D., Zukui L., Li Z., DiMaggio P.A., Garcia B.A., Floudas C.A. (2012). Novel Protein Identification Methods for Biomarker Discovery via a Proteomic Analysis of Periodontally Healthy and Diseased Gingival Crevicular Fluid Samples. J. Clin. Periodontol..

[35] Baliban R.C., Sakellari D., Li Z., Guzman Y.A., Garcia B.A., Floudas C.A. (2013). Discovery of biomarker combinations that predict periodontal health or disease with high accuracy from GCF samples based on high-throughput proteomic analysis and mixed-integer linear optimization. J. Clin. Periodontol..

[36] Choi Y.J., Heo S.H., Lee J.M., Cho J.Y. (2011). Identification of Azurocidin as a Potential Periodontitis Biomarker by a Proteomic Analysis of Gingival Crevicular Fluid. Proteome Sci..

[37] Carneiro L.G., Venuleo C., Oppenheim F.G., Salih E. (2012). Proteome Data Set of Human Gingival Crevicular Fluid From Healthy Periodontium Sites by Multidimensional Protein Separation and Mass Spectrometry. J. Periodontal Res..

[38] Carneiro L.G., Nouh H., Salih E. (2014). Quantitative Gingival Crevicular Fluid Proteome in Health and Periodontal Disease Using Stable Isotope Chemistries and Mass Spectrometry. J. Clin. Periodontol..

[39] Bostanci N., Heywood W., Mills K., Parkar M., Nibali L., Donos N. (2010). Application of Label-Free Absolute Quantitative Proteomics in Human Gingival Crevicular Fluid by LC/MS E (Gingival Exudatome). J. Proteome Res..

[40] Bostanci N., Ramberg P., Wahlander Å., Grossman J., Jönsson D., Barnes V.M., Papapanou P.N. (2013). Label-free quantitative proteomics reveals differentially regulated proteins in experimental gingivitis. J. Proteome Res..

[41] Huynh A.H., Veith P.D., McGregor N.R., Adams G.G., Chen D., Reynolds E.C., Ngo L.H., Darby I.B. (2015). Gingival Crevicular Fluid Proteomes in Health, Gingivitis and Chronic Periodontitis. J. Periodontal Res..

[42] Guzman Y.A., Sakellari D., Papadimitriou K., Floudas C.A. (2018). High-throughput proteomic analysis of candidate biomarker changes in gingival crevicular fluid after treatment of chronic periodontitis. J. Periodontal Res..

[43] Batschkus S., Cingoez G., Urlaub H., Miosge N., Kirschneck C., Meyer-Marcotty P., Lenz C. (2017). A new albumin-depletion strategy improves proteomic research of gingival crevicular flflfluid from periodontitis patients. Clin. Oral Investig..

[44] Marinho M.C., Pacheco A., Costa G., Ortiz N.D., Zajdenverg L., Sansone C. (2019). Quantitative gingival crevicular fluid proteome in type 2 diabetes mellitus and chronic periodontitis. Oral Dis..

[45] Silva-Boghossian C.M., Colombo A.P., Tanaka M., Rayo C., Xiao Y., Siqueira W.L. (2017). Quantitative proteomic analysis of gingival crevicular flflfluids from deciduous and permanent teeth. J. Clin. Periodontol..

[46] Odanaka H., Obama T., Sawada N., Sugano M., Itabe H., Yamamoto M. (2020). Comparison of protein profiles of the pellicle, gingival crevicular fluid, and saliva: Possible origin of pellicle proteins. Biol. Res..

[47] Colares V., Lima S., Sousa N., Araújo M.C., Pereira D., Mendes S., Teixeira S.A., Monteiro C.A., Bandeca M.C., Siqueira W.L. (2019). Hydrogen peroxide-based products alter inflammatory and tissue damage-related proteins in the gingival crevicular fluid of healthy volunteers: A randomized trial. Sci. Rep..

[48] Bostanci N., Belibasakis G.N. (2018). Gingival crevicular fluid and its immune mediators in the proteomic era. Periodontology 2000.

[49] Tsuchida S., Satoh M., Takiwaki M., Nomura F. (2018). Current Status of Proteomic Technologies for Discovering and Identifying Gingival Crevicular Fluid Biomarkers for Periodontal Disease. Int. J. Mol. Sci..

[50] Hutchens T.W., Yip T.T. (1993). New desorption strategies for the mass spectrometric analysis of macromolecules. Rapid Commun. Mass Spectrom..

[51] Pitteri S.J., Hanash S.M. (2007). Proteomic approaches for cancer biomarker discovery in plasma. Expert Rev. Proteom..

[52] Hodgetts A., Levin M., Kroll J.S., Langford P.R. (2007). Biomarker discovery in infectious diseases using SELDI. Future Microbiol..

[53] Whelan L.C., Power K.A., McDowell D.T., Kennedy J., Gallagher W.M. (2008). Applications of SELDI-MS technology in oncology. J. Cell. Mol. Med..

[54] Diamandis E.P. (2004). Mass spectrometry as a diagnostic and a cancer biomarker discovery tool: Opportunities and potential limitations. Mol. Cell. Proteom..

[55] Zhang X., Wei D., Yap Y., Li L., Guo S., Chen F. (2007). Mass spectrometry-based “omics” technologies in cancer diagnostics. Mass Spectrom. Rev..

[56] Semmes O.J., Feng Z., Adam B.L., Banez L.L., Bigbee W.L., Campos D., Cazares L.H., Chan D.W., Grizzle W.E., Izbicka E. (2005). Evaluation of serum protein profiling by surface-enhanced laser desorption/ionization time-of-flight mass spectrometry for the detection of prostate cancer: I. Assessment of platform reproducibility. Clin. Chem..

[57] Savino R., Terracciano R. (2012). Mesopore-assisted profiling strategies in clinical proteomics for drug/target discovery. Drug Discov. Today.

[58] Mann M., Hendrickson R.C., Pandey A. (2001). Analysis of proteins and proteomes by mass spectrometry. Annu. Rev. Biochem..

[59] Cho Y.T., Su H., Huang T.L., Chen H.C., Wu W.J., Wu P.C., Wu D.C., Shiea J. (2013). Matrix-assisted laser desorption ionization/time-of-flight mass spectrometry for clinical diagnosis. Clin. Chim. Acta.

[60] Terracciano R., Pasqua L., Casadonte F., Frascà S., Preianò M., Falcone D., Savino R. (2009). Derivatized mesoporous silica beads for MALDI-TOF MS profiling of human plasma and urine. Bioconjug. Chem..

[61] Savino R., Paduano S., Preianò M., Terracciano R. (2012). The proteomics big challenge for biomarkers and new drug-targets discovery. Int. J. Mol. Sci..

[62] Greco V., Piras C., Pieroni L., Ronci M., Putignani L., Roncada P., Urbani A. (2018). Applications of MALDI-TOF mass spectrometry in clinical proteomics. Expert Rev. Proteom..

[63] Terracciano R., Casadonte F., Pasqua L., Candeloro P., Di Fabrizio E., Urbani A., Savino R. (2010). Enhancing plasma peptide MALDI-TOF-MS profiling by mesoporous silica assisted crystallization. Talanta.

[64] Tsutsumi-Ishii Y., Hasebe T., Nagaoka I. (2000). Role of CCAAT/enhancer-binding protein site in transcription of human neutrophil peptide-1 and -3 defensin genes. J. Immunol..

[65] Gibb S., Strimmer K. (2015). Differential protein expression and peak selection in mass spectrometry data by binary discriminant analysis. Bioinformatics.

[66] Bellagambi F.G., Degano I., Ghimenti S., Lomonaco T., Dini V., Romanelli M., Mastorci F., Gemignani A., Salvo P., Fuoco R. (2018). Determination of salivary α-amylase and cortisol in psoriatic subjects undergoing the Trier Social Stress Test. Microchem. J..

[67] Lomonaco T., Ghimenti S., Biagini D., Bramanti E., Onor M., Bellagambi F.G., Fuoco R., Di Francesco F. (2018). The effect of sampling procedures on the urate in oral fluid and lactate concentration in oral fluid. Microchem. J..

[68] Aebersold R., Mann M. (2003). Mass spectrometry-based proteomics. Nature.

[69] Whitehouse C.M., Dreyer R.N., Yamashita M., Fenn J.B. (1985). Electrospray interface for liquid chromatographs and mass spectrometers. Anal. Chem..

[70] Douglas D.J., Frank A.J., Mao D. (2005). Linear ion traps in mass spectrometry. Mass Spectrom. Rev..

[71] Nolting D., Malek R., Makarov A. (2019). Ion traps in modern mass spectrometry. Mass Spectrom. Rev..

[72] Hu Q., Noll R.J., Li H., Makarov A., Hardman M., Graham Cooks R. (2005). The Orbitrap: A new mass spectrometer. J. Mass Spectrom..

[73] Michalski A., Damoc E., Lange O., Denisov E., Nolting D., Müller M., Viner R., Schwartz J., Remes P., Belford M. (2012). Ultra high resolution linear ion trap Orbitrap mass spectrometer (Orbitrap Elite) facilitates top down LC MS/MS and versatile peptide fragmentation modes. Mol. Cell. Proteom..

[74] Vitorino R. (2018). Digging Deep into Peptidomics Applied to Body Fluids. Proteomics.

[75] Maes E., Dyer J.M., McKerchar H.J., Deb-Choudhury S., Clerens S. (2017). Protein-protein cross-linking and human health: The challenge of elucidating with mass spectrometry. Expert Rev. Proteom..

[76] Wang Y., Griffiths W.J., Jörnvall H., Agerberth B., Johansson J. (2002). Antibacterial peptides in stimulated human granulocytes: Characterization of ubiquitinated histone H1A. Eur. J. Biochem..

[77] Theilgaard-Mönch K., Jacobsen L.C., Nielsen M.J., Rasmussen T., Udby L., Gharib M., Arkwright P.D., Gombart A.F., Calafat J., Moestrup S.K. (2006). Haptoglobin is synthesized during granulocyte differentiation, stored in specific granules, and released by neutrophils in response to activation. Blood.

[78] Weisel J.W. (2005). Fibrinogen and fibrin. Adv. Protein Chem..

[79] Philp D., Goldstein A.L., Kleinman H.K. (2004). Thymosin beta4 promotes angiogenesis, wound healing, and hair follicle development. Mech. Ageing Dev..

[80] Reti R., Kwon E., Qiu P., Wheater M., Sosne G. (2008). Thymosin beta4 is cytoprotective in human gingival fibroblasts. Eur. J. Oral Sci..

[81] Wei L., Liu M., Xiong H. (2019). Role of Calprotectin as a Biomarker in Periodontal Disease. Mediat. Inflamm..

[82] Terracciano R., Pelaia G., Preianò M., Savino R. (2015). Asthma and COPD proteomics: Current approaches and future directions. Proteom. Clin. Appl..

[83] Chapple I.L., Brock G., Eftimiadi C., Matthews J.B. (2002). Glutathione in gingival crevicular fluid and its relation to local antioxidant capacity in periodontal health and disease. Mol. Pathol..

[84] Lim S.Y., Raftery M.J., Goyette J., Geczy C.L. (2010). S-glutathionylation regulates inflammatory activities of S100A9. J. Biol. Chem..

[85] Dale B.A., Fredericks L.P. (2005). Antimicrobial peptides in the oral environment: Expression and function in health and disease. Curr. Issues Mol. Biol..

[86] Sakamoto N., Mukae H., Fujii T., Ishii H., Yoshioka S., Kakugawa T., Sugiyama K., Mizuta Y., Kadota J., Nakazato M. (2005). Differential effects of alpha- and beta-defensin on cytokine production by cultured human bronchial epithelial cells. Am. J. Physiol. Lung Cell. Mol. Physiol..

[87] Panyutich A.V., Hiemstra P.S., van Wetering S., Ganz T. (1995). Human neutrophil defensin and serpins form complexes and inactivate each other. Am. J. Respir. Cell Mol. Biol..

[88] Gursoy U.K., Könönen E. (2012). Understanding the roles of gingival beta-defensins. J. Oral Microbiol..

[89] Türkoğlu O., Emingil G., Kütükçüler N., Atilla G. (2009). Gingival crevicular fluid levels of cathelicidin LL-37 and interleukin-18 in patients with chronic periodontitis. J. Periodontol..

[90] Janciauskiene S., Zelvyte I., Jansson L., Stevens T. (2004). Divergent effects of alpha1-antitrypsin on neutrophil activation, in vitro. Biochem. Biophys. Res. Commun..

[91] Subramaniyam D., Glader P., von Wachenfeldt K., Burneckiene J., Stevens T., Janciauskiene S. (2006). C-36 peptide, a degradation product of alpha1-antitrypsin, modulates human monocyte activation through LPS signaling pathways. Int. J. Biochem. Cell Biol..

[92] Blaurock N., Schmerler D., Hünniger K., Kurzai O., Ludewig K., Baier M., Brunkhorst F.M., Imhof D., Kiehntopf M. (2016). C-Terminal Alpha-1 Antitrypsin Peptide: A New Sepsis Biomarker with Immunomodulatory Function. Mediat. Inflamm..

[93] Socransky S.S., Haffajee A.D., Cugini M.A., Smith C., Kent R.L. (1998). Microbial complexes in subgingival plaque. J. Clin. Periodontol..

[94] Lamster I.B., Ahlo J.K. (2007). Analysis of gingival crevicular fluid as applied to the diagnosis of oral and systemic diseases. Ann. N. Y. Acad. Sci..

[95] Preianò M., Pasqua L., Gallelli L., Galasso O., Gasparini G., Savino R., Terracciano R. (2012). Simultaneous extraction and rapid visualization of peptidomic and lipidomic body fluids fingerprints using mesoporous aluminosilicate and MALDI-TOF MS. Proteomics.

[96] Albalat A., Stalmach A., Bitsika V., Siwy J., Schanstra J.P., Petropoulos A.D., Vlahou A., Jankowski J., Persson F., Rossing P. (2013). Improving peptide relative quantification in MALDI-TOF MS for biomarker assessment. Proteomics.

[97] Hillenkamp F., Peter-Katalinic J. (2007). MALDI MS: A Practical Guide to Instrumentation, Methods and Applications.

[98] Szájli E., Fehér T., Medzihradszky K.F. (2008). Investigating the Quantitative Nature of MALDI-TOF MS. Mol. Cell. Proteom..

[99] Bucknall M., Fung K.Y., Duncan M. (2002). Practical quantitative biomedical applications of MALDI-TOF mass spectrometry. J. Am. Soc. Mass Spectrom..

[100] Urso E., Le Pera M., Bossio S., Sprovieri T., Qualtieri A. (2010). Quantification of thymosin beta(4) in human cerebrospinal fluid using matrix-assisted laser desorption/ionization time-of-flight mass spectrometry. Anal. Biochem..

[101] Xu H., Liu M., Huang X., Min Q., Zhu J.J. (2018). Multiplexed Quantitative MALDI MS Approach for Assessing Activity and Inhibition of Protein Kinases Based on Postenrichment Dephosphorylation of Phosphopeptides by Metal-Organic Framework-Templated Porous CeO_2_. Anal. Chem..

[102] Guitot K., Scarabelli S., Drujon T., Bolbach G., Amoura M., Burlina F., Jeltsch A., Sagan S., Guianvarc’h D. (2014). Label-free measurement of histone lysine methyltransferases activity by matrix-assisted laser desorption/ionization time-of-flight mass spectrometry. Anal. Biochem..

[103] Tucher J., Somasundaram P., Tholey A., Cramer R. (2016). Quantitative MALDI MS Using Ionic Liquid Matrices in Advances. MALDI and Laser-Induced Soft Ionization Mass Spectrometry.

[104] Deng H., Xu Y., Zhang L., Sethala R., Zhang L. (2009). Proteomic analysis in drug discovery. Handbook of Drug Screening.

[105] Terracciano R., Preianò M., Maggisano G., Pelaia C., Savino R. (2019). Hexagonal Mesoporous Silica as a Rapid, Efficient and Versatile Tool for MALDI-TOF MS Sample Preparation in Clinical Peptidomics Analysis: A Pilot Study. Molecules.

[106] Zhang J., Xin L., Shan B., Chen W., Xie M., Yuen D., Zhang W., Zhang Z., Lajoie G.A., Ma B. (2012). PEAKS DB: De novo sequencing assisted database search for sensitive and accurate peptide identification. Mol. Cell. Proteom..

[107] Ma B., Zhang K., Hendrie C., Liang C., Li M., Doherty-Kirby A., Lajoie G. (2003). PEAKS: Powerful software for peptide de novo sequencing by tandem mass spectrometry. Rapid Commun. Mass Spectrom..

[108] Budamgunta H., Olexiouk V., Luyten W., Schildermans K., Maes E., Boonen K., Menschaert G., Baggerman G. (2018). Comprehensive Peptide Analysis of Mouse Brain Striatum Identifies Novel sORF-Encoded Polypeptides. Proteomics.

[109] Taylor J.J., Preshaw P.M. (2016). Gingival crevicular fluid and saliva. Periodontology 2000.

